# Service Quality in Early Intervention Centres: An Analysis of Its Influence on Satisfaction and Family Quality of Life

**DOI:** 10.3390/children8080716

**Published:** 2021-08-21

**Authors:** Inmaculada-Concepción Jemes-Campaña, Rita-Pilar Romero-Galisteo, Pablo Gálvez-Ruiz, Maria-Teresa Labajos-Manzanares, Noelia Moreno-Morales

**Affiliations:** 1 Doctoral School, University of Málaga, 29071 Málaga, Spain; ijemes@uma.es; 2 Department of Fhysiotherapy, University of Málaga, C/Arquitecto Francisco Peñalosa, s/n., 29071 Málaga, Spain; mtlabajos@uma.es (M.-T.L.-M.); nmm@uma.es (N.M.-M.); 3 Faculty of Law and Social Sciences, Valencian International University, 46002 Valencia, Spain; pgalvez@universidadviu.com

**Keywords:** Early Intervention Centres, perceived quality of service, family quality of life, satisfaction

## Abstract

Early Intervention (EI) is a set of interventions focused on responding to the needs of children with or at risk of developmental problems. This study aimed to investigate the relationships between the perceived quality of service, satisfaction and family quality of life. Methods: to conduct a multi-centre, transversal study with a non-probabilistic sample. The participants (N = 1551) were families from 24 Early Intervention Centres (EICs) located in Spain. Results: The results indicated an adequate fit of the measurement and structural models, with the latter showing a capacity of 73% to predict the family quality of life. The structural model established that the perceived service quality was a positive and significant predictor of satisfaction (β = 0.85; *p* < 0.001). Both the perceived quality of service (β = 0.28; *p* < 0.001) and satisfaction (β = 0.33; *p* < 0.001) obtained a similar positive and significant relationship with family quality of life, which was slightly stronger than satisfaction. Conclusions: This study provided a better understanding of the importance of the services offered in EICs and their influence on the satisfaction and family quality of life of their users. Thus, delving into these relationships was highly relevant for decision-making in the context of EI.

## 1. Introduction

Early Intervention (EI) is a set of interventions focused on responding to the transient or permanent needs of children between 0 and 6 years of age with or at risk of suffering with developmental disorders [1].

The main objective of EI is to favour the development and wellbeing of a child and his/her family, facilitating his/her integration in the environment, as well as his/her personal autonomy [1]. EI centres work on the cognitive area; language and communication, personal autonomy and motor skills; and also provide counselling and individual and/or group intervention with families [2].

Although EI programmes have traditionally disregarded environmental and family contexts [3], this approach has changed in the last decades towards a model that values the family scope to a greater extent [4]. The growing interest in the family and a child’s environment is due to their relevance in the evaluation and rehabilitation of children with special needs, as they play an essential role throughout the entire process [5]. Moreover, different studies support the participation and the empowerment of the family and a rich environment to promote advancements in the development of children with developmental disorders [6,7,8,9,10].

On the other hand, EICs, as organisations related to services, must focus their management strategies on the client and his/her needs. Ensuring these objectives is necessary to facilitate the early access to EICs and to streamline the acceptance process to these services [11]. In this sense, it is essential to provide good service, and the evaluation of such service is a basic step in the development of high-quality improvement programmes.

Access to EI services in Spain varies depending on the autonomous community in which it is provided [12]. Although developmental alterations or their risks can be detected in healthcare services, and in school or family environments, referral to the EI service can be completed through specialised Social Services, Education Services or, as is the case in Andalusia, through EI units of the Health Service [13].

Regarding human resources, the professional profiles of the EI resources depend on the autonomous community and the means of funding, with psychologists, speech therapists and physiotherapists being the most common, followed by special education teachers, occupational therapists and other child development professionals [14].

In the case of EI, the perspective of the parents about the quality of the service received can facilitate changes in such services, thus maximising the opportunities for psychomotor development in children with developmental disorders. Given the importance of this aspect, it is relevant to know and understand the beliefs of the families regarding the quality of the EI services, as well as the expectations, engagement and perspectives of the families [15,16,17]. Due to the multidimensionality of the construct, as well as the lack of reliability and operationalisation, several authors highlight the need to adapt the dimensions of such a model to the context and service to be evaluated [18], since, depending on the type of business, the dimensions of service quality can be considerably different.

In the evaluation of social services, Moliner-Cantos et al. [19] state that service quality contributes to improving the conditions of social services and favours the quality of life of its users, an approach that has drawn the attention of different studies in the context of EI [20]. Moreover, the study of the quality of life could serve as a guide in a holistic approach to the evaluation and intervention of EIC and help to plan interventions for both children and their parents/guardians [21]. Aside from the quality of life of the child, the quality of life of the family can also shed light on the efficacy and quality of the EI programme that they receive [22].

In fact, in the last decade, family quality of life has become a decisive element for the evaluation of services provided in EI, since identifying the variables that improve quality of life can determine the appropriate treatment [23]. Several studies demonstrate the relationship between both constructs, with service quality being a predictor of the quality of life [24].

Concerning satisfaction, this construct has gained an increased interest in the scientific literature for being considered as preceding loyalty [25], which is a fundamental objective in service organisations. Specifically in EI, some studies have evaluated the levels of satisfaction [26] and the quality of life [27] of families who use these services, although they do not delve into the relationship between the two constructs. In the hospital context, better levels of quality of life are detected in hospitalised patients when their satisfaction with the service is high [28]. The study by Lanfredi et al. [29] in a mental health population reported similar results, asserting that satisfaction is a precursor to quality of life.

Satisfaction and service quality have also shown a direct and positive correlation in different studies. Such investigations state that service quality precedes user satisfaction [30] and, despite the lack of thorough research on this relationship in the EI context, such a correlation has been investigated in other contexts, such as sports [31] and transportation [32].

**Figure 1:**
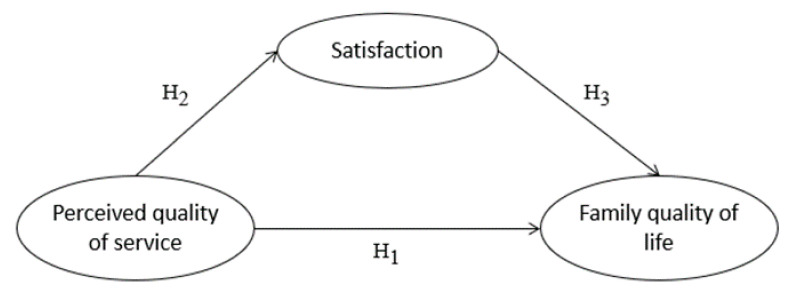
Proposed conceptual model.

Therefore, the aim of this study was to determine the influence of perceived service quality on family quality of life (H_1_) and satisfaction (H_2_), as well as the influence of satisfaction on family quality of life (H_3_) (Figure 1):

## 2. Materials and Methods

### 2.1. Participants

This was a quantitative study with a non-probabilistic sample of 1551 parents (1235 women and 308 men) from 24 EIC located in the region of Andalusia (Southern Spain). Early intervention services in Spain were organised differently in each of the autonomous communities into which the country is divided, and each of these had the authority to organise the services that it provided in a different way [33]. In the specific case of Andalusia, it was legislated according to Decree 85/2016, of 26 April [13], which regulates the integral intervention of Early Childhood Care. The participants were recruited during the period from October to November 2018. The inclusion criteria were: (1) family members and/or caregivers of children with or at risk of presenting developmental disorders/developmental delays who attended EI centres; (2) family members and/or caregivers with literacy skills in Spanish to understand the questionnaire; and (3) family members and/or caregivers who provided a duly completed informed consent form. Participants were not excluded on the basis of the type of injury their child suffered or previous hospital care received.

At the beginning of the study, the managers of the different EI centres were contacted via e-mail. The questionnaires were sent to these centres via ordinary mail and identified with a numerical code. Authorisation was requested from the different centres through a letter explaining the purpose of the investigation and the procedure to be carried out. The participations received verbal and written information on the objectives and methodology of the study, and they freely agreed to collaborate by signing an informed consent form. In accordance with the Declaration of Helsinki, the anonymity and confidentiality of their responses was assured. The questionnaires took around 15 min to complete and were completed in the waiting rooms of the EIC; the participants were asked for maximum sincerity and honesty.

Permission to undertake this study was granted by the Research Ethics Committee of the University of Málaga (code 22-2018-H). Table 1 summarises the background characteristics of the sample.

### 2.2. Measures

The participants answered three questionnaire items. To measure the perceived quality of EICs, the Inventory of Quality in Early Intervention Centres (IQEIC) [34] was used, composed of four dimensions: centre facilities (CF), treatment room and material (TRM), qualified staff (QS), and technical or specific information (TSI). This tool was used in previous studies, obtaining adequate psychometric properties in the adjustment of the model (χ^2^/df = 2.53; CFI = 0.94; TLI = 0.92; IFI = 0.94; RMSEA = 0.059). The response format for all items was a 5-point Likert scale rated from 1 (*strongly disagree*) to 5 (*strongly agree*). To measure the family quality of life, the CdVF-ER for families with children under the age of 18 years [35] was used, divided into five dimensions: Family Resources (FR), Family adaptation (FA), Family Climate (FC), Emotional Stability (ES), and Economic Well-Being (EW-B). The instrument was answered with a 5-point Likert scale with the following answer choices: never, rarely, sometimes, often, and always. Furthermore, the option, *not applicable,* was also present for the families to select if the item did not reflect their situation. Finally, a satisfaction scale composed of three items was used [36], which had already been employed in this context [37] obtaining an adequate internal consistency (α = 0.90), with the same response format as the IQEIC instrument. Appendix A shows the items used for each scale.

### 2.3. Statistical Procedures

Descriptive statistics were calculated for the participants’ characteristics (*n* [%]). All the data were analysed using SPSS Statistics and AMOS Graphics statistical software (version 21.0). An exploratory factor analysis (EFA) was conducted using principal component analysis and oblimin oblique rotation. The choice of this experimental route was based on the fact that this study employed a measurement model composed of three constructs that had not been previously validated. Assumptions needed for factor analysis were verified and, subsequently, the factorability of the items was examined via Bartlett’s sphericity test and the Kaiser-Meyer-Olkin (KMO) measure of sampling adequacy. To analyse the internal consistency, Cronbach’s alpha and the corrected homogeneity index (CHI) were calculated. In order to test the model we used the classical two-step assessment proposed by different authors [38,39] using a maximum likelihood method of estimation recommended by Jöreskog and Sörbom [40]: (1) a confirmatory factor analysis (CFA) was conducted to verify the EFA structure and assess the measurement model, and (2) a structural equation model (SEM) analysed the predicted hypothesised relationships between the three reflective variables: perceived service quality, family quality of life, and satisfaction. Convergent validity and reliability were determined by assessing the composite reliability (CR) and the average variance extracted (AVE). According to Hair et al. [41], CR values over 0.70 and AVE scores over 0.50 were considered as good scores; however, AVE values slightly below 0.50 were also acceptable as long as the CR measured more than 0.60 [42].

Before the main analysis, evidence of potential multicollinearity between predictors was verified using bivariate comparisons. Looking at the correlations among the predictors, we found that the highest correlation was found between the service quality and satisfaction (0.78) without multicollinearity problems, according to the established values (r = 0.90) [43]. We also examined the variance inflation factor (VIF) for our final model, obtaining a maximum value of 1.86, lower than the established values in which multicollinearity was considered problematic for the estimates [44]

The following goodness-of-fit indexes were used in both analyses to test the adequacy of the models: the ratio of chi-square to its degrees of freedom (χ^2^/df), the Comparative Fit Index (CFI), the Tucker–Lewis Index (TLI), the Incremental Fit Index (IFI), the Parsimony Comparative Fit Index (PCFI), and the Root Mean Square Error of Approximation (RMSEA). For these indexes, the following cut-off values were adopted: χ^2^/df ≤ 5 [45], CFI, TLI and IFI ≥ 0.90 [46], PCFI ≥ 0.80 [47] and RMSEA ≤ 0.08 [48,49].

## 3. Results

### 3.1. Exploratory Factor Analysis and Reliability

In terms of structural validity, the analysis of the measurement model yielded a KMO measure of 0.94 with a significant Bartlett’s test (*p* < 0.001). The inspection of eigenvalues produced a structure accounting for 56.63% of the variance. The items with communalities above 50% and items with factor loadings over the minimum acceptable value (0.50) were accepted [41]. Thus, the IQEIC maintained the original item structure, but the results of the CdVF-ER suggested a different dimensionality, with a factor structure of 21 items. With respect to reliability, high alpha coefficients (α = 0.70) [50] demonstrated a good internal consistency for the different factors of IQEIC (CF = 0.72; TRM = 0.88; QS = 0.94; TSI = 0.89), CdVF-ER (FC = 0.75; ES = 0.77; EW-B = 0.74; FA = 0.77; FR = 0.71) and satisfaction (0.93).

### 3.2. Confirmatory Factor Analysis

The measurement model of the 10-factor structure was examined, yielded the corresponding value of: χ^2^(df = 1130) = 5456.81; χ^2^/df = 4.82; CFI = 0.90; TLI = 0.89; IFI = 0.90; PCFI = 0.83; and RMSEA = 0.050. An examination of the factor loading (λ) and modification indices (MI) indicated the possible areas for improvement. In all cases, the observed variables showed a factor loading value of greater than the conservative threshold of 0.50 [41]. According to the MI, with the assumption that all the respective items involved presented some similar content, covariance was established as being between CF1 and CF2 (MI = 186.27), TRM1 and TRM2 (MI = 264.72), QS1 and QS2 (MI = 134.14), and TSI5 and TSI6 (MI = 225.89). Following the inclusion of the four covariance, there was an improvement in the indices of fit: χ^2^(df = 1122) = 3896.07; χ^2^/df = 3.47; CFI = 0.93; TLI = 0.93; IFI = 0.94; PCFI = 0.85; and RMSEA = 0.40. All indices were satisfactory, including the value of χ^2^/df, despite its high sensitivity to the sample size [41]. Construct reliabilities were greater than 0.60, average variance extracted (AVE) values were adequate [42] assuming the convergent validity, and squared interconstruct correlations were smaller than the respective AVE values [42] accepting the discriminant validity (Table 2).

### 3.3. Structural Equation Assessment

Once the reliability and suitability of the measurement model were confirmed, the hypothetical model and the standardised regression loadings were subjected to a causal path analysis. The results indicated support for all the causal relationships, along with an adequate goodness-of-fit for the causal model: χ^2^(df = 1064) = 5251.84; χ^2^/df = 4.93; NFI = 0.91; CFI = 0.92; TLI = 0.90; IFI = 0.91; and RMSEA = 0.50. The hypothetical model established that the perceived service quality was positive and the significant predictors of satisfaction (β = 0.85; *p* < 0.001), illustrating a predictive capacity of 73%. Both perceived that service quality (β = 0.43; *p* < 0.001) and satisfaction (β = 0.48; *p* < 0.001) obtained a similar positive and significant relationship with family quality of life, which was slightly higher for satisfaction. However, the obtained results show that this was a partial mediation between the perceived service quality and family quality of life, governed by satisfaction (Table 3).

## 4. Discussion

The aim of this research was to analyse the relationship between the perceived service quality, family quality of life and satisfaction in the context of EIC. The findings show that the perceived service quality can be used to predict the family quality of life in children who receive EI services, and that satisfaction is a variable that mediates family quality of life.

In general, EICs that provide services to children and their parents, are aware of the increasing interest in measuring the family quality of life, received support and perceptions of their users [51]. In this sense, different studies have delved into these constructs, although in an isolated manner, without relating the influence among them [21,52,53]. Nowadays, the field of EI is undergoing a significant conceptual change, and the old intervention model is being replaced with a social model in which the family and the environment are fundamental axes [1], thus the evaluation of aspects related to them is especially relevant.

There is evidence of the need to study family quality of life [54], perceived service quality [55] and satisfaction [56] in EI. In this sense, it is important to note that the use of valid and reliable measurement tools is a key component in both clinical practice and research. Therefore, the evaluation of the psychometric properties of the measurement instruments will help researchers and clinicians to choose the most suitable tool in order to better evaluate the construct they wish to assess [57], and, in this line, the present study contributes to the existing literature by proposing an evaluation model for users of EIC. The analyses conducted in this study show the same factor structure for the IQEIC, however, for the CdVF-ER the results show a scale with less items compared to Giné et al. [35]. The resulting measurement model consists of 50 items: 26 for service quality, 21 for family quality of life and 3 for satisfaction. For this model, the relevance criteria of the factor analysis are satisfactorily met, obtaining significance in the Bartlett test and KMO indices higher than 0.90, with an explained variance extracted higher than 50%. In addition, the internal consistency measured through Cronbach’s alpha coefficient is adequate. Regarding the validity of the model, a satisfactory fit in the confirmatory factor analysis, and revealed good reliability evidence in terms of internal consistency and composite reliability. Furthermore, the validity of the model is demonstrated through the average variance extracted and the discriminant validity. Therefore, it is an optimal model for measuring the adequate psychometric properties that can be rapidly applied. In this regard, it is found that the factorial structure and the analysis of internal consistency give the instrument a validity and reliability that makes it suitable for use in the context of EI.

With respect to the relationships model, all hypotheses are statistically significant and the model presents an acceptable fit to the data, thus confirming the three hypotheses. The results proved that the perceived service quality has a positive and significant influence on family quality of life, which supports H_1_, in line with Moliner-Cantos et al. [19] in the context of social services. It also shows a strong positive and significant influence on satisfaction, which supported H_2_, in agreement with studies conducted in other contexts, such as hospitality [58] and tourism [59]. Almasri et al. [60] analysed the relationship between service quality and family wellbeing in a cerebral palsy population, obtaining positive and significant relationships between these two constructs. Therefore, despite the fact that the direct focus is not family quality of life, it is shown that there is a growing interest in the study of the service provided and the family. An example of this is the work of Park and Kim [61], who analysed the relationship between the degree of motor deterioration of a child with developmental disorders and family stress and depression, or the study of Colver et al. [62], who explored the degree of disability and pain in the participation of the child with cerebral palsy in the home environment. Lastly, regarding satisfaction (H_3_), it is observed that this variable has a positive and significant influence on family quality of life, which is in agreement with the results obtained by Lanfredi et al. [29] in a sample of schizophrenia patients.

One of the limitations of this study is the sample of participants, which is not representative of the EICs of Spain. However, the absence of common legislation in the entire Spanish territory favours the disparity of management models, which hinders the generalisation of the results of this investigation to other contexts. Another limitation is related to the questionnaire used to evaluate the perceived quality of the service, since it is designed to evaluate the EI service in the specialised centres by gathering information about tangible aspects, such as the facilities, treatment rooms and materials. Therefore, the reliability and validity that this tool has for EI centres is not guaranteed for those EI services provided only in natural environments, such as the family home.

Consequently, this study provides an evaluation model for family quality of life, highlighting the importance of perceived service quality and satisfaction through structural equation modeling. Despite the novelty of this type of analysis in EI, recent studies have used this methodology to evaluate how family quality of life is influenced by the quality of the relationship between the family and the reference professional [63,64], and how the support received by the families from the professionals of EICs influences their quality of life [24]. Therefore, we consider that this study provides an interesting contribution to the specialised academic literature and paves the way for new research lines with the use of this methodology in the context of populations with functional diversity, such as the exploration into the influence of the degree of dependency of the child with developmental disorders on the perception of service quality, satisfaction and family quality of life.

Following the approach proposed by Ashcraft et al. [10], the current study contributes to a better understanding of the mechanisms by which parents can achieve empowerment and the expected outcomes in the field of pediatric rehabilitation in general, and EI in particular.

Regularly integrating assessments of perceived service quality in EI centres with instruments validated in this field/sector provides detailed information about the strengths and weaknesses of EI centres. In this way, managers/directors and professionals of EI centres can focus their efforts on maintaining factors that score higher and improving factors where scores are lower. This could lead to an increase in the quality of the service offered in EI centres.

## 5. Conclusions

The perceived quality and satisfaction with EICs can become important tools to achieve family quality of life, both in the children and families who receive EI services. This study delves into the relationships between these aspects, obtaining results of great relevance considering the lack of similar studies in the context of EI. Thus, the contributions of the present study allow providers of these services to respond to changing needs in a flexible manner, paving the way for future research.

## Figures and Tables

**Table 1:** Sociodemographic characteristics of the participants.

Characteristics	*n*	%	Missing (%)
Gender			8 (0.6)
Male	308	19.8	
Female	1235	79.6	
Age (years)			129 (8.5)
20–30	201	12.9	
31–40	847	54.9	
41–50	338	21.7	
>51	36	2.3	
Academic achievement			119 (7.6)
Elementary education	74	4.8	
Middle school completion	311	20.1	
High school education	562	36.2	
College education	433	27.9	
Master or doctorate graduates	52	3.4	
Length of stay (in months)			153 (10.2)
0–12	643	41.4	
13–24	459	29.5	
25–36	200	12.8	
>37	96	6.1	

**Table 2:** Cronbach Alpha (α), composite reliability (CR), average variance extracted (AVE) and discriminant validity.

Factors	α	CR	AVE	FC	ES	EW-B	FA	FR	CF	TRM	QS	TSI	SAT
FC	0.75	0.84	0.47	1.0									
ES	0.77	0.77	0.53	0.11	1.0								
EW-B	0.74	0.82	0.48	0.29	0.23	1.0							
FA	0.77	0.81	0.52	0.07	0.31	0.12	1.0						
FR	0.71	0.75	0.50	0.03	0.28	0.04	0.16	1.0					
CF	0.72	0.84	0.47	0.01	0.01	0.02	0.01	0.01	1.0				
TRM	0.88	0.89	0.58	0.09	0.01	0.02	0.01	0.01	0.71	1.0			
QS	0.94	0.95	0.68	0.01	0.03	0.02	0.01	0.01	0.31	0.38	1.0		
TSI	0.89	0.89	0.58	0.01	0.06	0.03	0.02	0.01	0.40	0.44	0.49	1.0	
SAT	0.93	0.93	0.82	0.01	0.01	0.03	0.02	0.34	0.37	0.62	0.62	0.72	1.0

Note. FC = family climate; ES = emotional stability; EW-B = economic well-being; FA = family adaptation; FR = family resources; CF = centre facilities; TRM = treatment room and material; QS = qualified staff; TSI = technical or specific information; and SAT = satisfaction.

**Table 3:** Standardised regression weights for the causal paths.

Relationships	Hypothesised Relationship	Standardised Coefficient	Results
Perceived quality of service → Quality of family life	H_1_	0.28 ***	Supported
Perceived quality of service → satisfaction	H_2_	0.85 ***	Supported
Satisfaction → Quality of family life	H_3_	0.33 ***	Supported

Note. *** *p*-value < 0.001.

## Data Availability

The data that support the findings of this study are available from the corresponding author, Rita Pilar Romero-Galisteo, upon reasonable request.

## Appendix A

**Table A1:** Survey items.

	Inventory of Quality in Early Intervention Centres (IQEIC) [37]
	Centre facilities
CF1	The centre is well located geographically
CF2	It is easy to reach the centre by public transport
CF3	The cleaning of the centre is adequate
CF4	The lighting of the centre is adequate
CF5	The waiting room is comfortable
CF6	The number of chairs in the waiting room is enough
	Treatment rooms and material
TRM1	The number of treatment rooms is enough
TRM2	The treatment rooms are large enough
TRM3	The materials that are used in the centr are suitable
TRM4	The materials are in good condition for use
TRM5	The materials that are used in the treatment rooms are safe
TRM6	The work materials comply with the health and hygiene conditions
	Qualified staff
QS1	The attention that users receive at the centre is suitable
QS2	Qualified staff have the necessary knowledge
QS3	Qualified staff are accessible
QS4	Qualified staff are available when users need them
QS5	Qualified staff have a close treatment
QS6	I value the contributions and initiatives of the qualified staff
QS7	Qualified staff are coordinated among themselves
QS8	Qualified staff know to adapt tasks to the user’s needs
	Technical or specific information
TSI1	The activities carried out with the user seem appropriate
TSI2	The activities proposed for users to work on at home are feasible
TSI3	The information received at the beginning of the treatment is consistent with the tasks subsequently performed
TSI4	I usually receive some programs to work with the user
TSI5	I usually receive some report about the progression of the user
TSI6	The information received about the user is clear
	Quality of life (CdVF-ER)
	Family Resources
FR1	My family adapts to the needs of the relative with developmental disorder
FR2	Our relatives and friend understand that the relative with developmental disorder can have a different behaviour
FR3	My family organises considering the needs of the relative with developmental disorder
	Family Adaptation
FA1	My family respects the small decisions made by the relative with developmental disorder (choose clothes, organise his/her space, etc.)
FA2	The relative with developmental disorder respects others when walking down the street
FA3	The relative with developmental disorder is physically autonomous
FA4	The relative with developmental disorder has a good relationship with his/her classmates
	Family Climate
FC1	All the members of my family trust each other enough to ask for help whenever they need it
FC2	All the members of my family show affection and love
FC3	My family adequately solves the conflicts that may arise among us
FC4	All the members of my family can pour out their hearts whenever they need to
FC5	All the members of my family talk openly about our concerns
	Emotional Stability
ES1	My family feels good when we see the relative with developmental disorder
ES2	My family is at peace, because we see that the relative with developmental disorder is progressing
ES3	The relative with developmental disorder has material goods suitable for his/her age (games, daily use material, etc)
	Economic Well-Being
EW-B1	My family can afford the needs of all its members
EW-B2	My family has enough resources to overcome critical and difficult moments
EW-B3	My family can afford the psychological and/or psychiatric attention of all its members
EW-B4	My family can afford some craving
EW-B5	My family can cover the costs derived from the participation of the relative with developmental disorder in social leisure activities
EW-B6	My family is in enough economic balance to face the future without worries
	Satisfaction [39]
SAT1	I am satisfied with the service provided in this centre
SAT2	I am happy with that this centre offers me
SAT3	My decision to go to this centre was appropriate
